# Medical Examining Board

**Published:** 1877-04-20

**Authors:** 


### MEDICAL EXAMINING BOARD.

Complaints reach us that in many places druggists and drug clerks are prescribing for patients. It is not necessary for us to say that in the State of Georgia this is unlawful. They are not even authorized to sell drugs without license from the Examining Board of the State, and heavy penalties attach to the violation of this law. Doctors who hold diplomas from legitimate Institutions are authorized to practice and to sell drugs without license from the State Board. But practitioners not graduates, can neither sell drugs nor practice medicine in Georgia, without such license. Nor are certain old women and negroes, as is the case in this city, authorized to practice medicine. It is not, however, incumbent upon the Board to hunt up and prosecute these cases, but it is the duty of every good citizen to report such violations, and judges of courts should charge the juries in reference to this, as to other encroachments upon the laws of the State. P.

